# Supplementary material for: Association between oral microbiome and seven types of cancers in East Asian population: a two-sample Mendelian randomization analysis
Source: Front Mol Biosci. 2023 Nov 21;10:1327893. doi: 10.3389/fmolb.2023.1327893 (PMC10702768; doi:10.3389/fmolb.2023.1327893)
Supplement: Supplementary file 1 [file DataSheet1.ZIP › Supplementary Materials/MR plots of saliva/saliva═╝/Pancreatic cancer/pheno.2269_to_pancreatic cancer_leave_one_out.pdf]

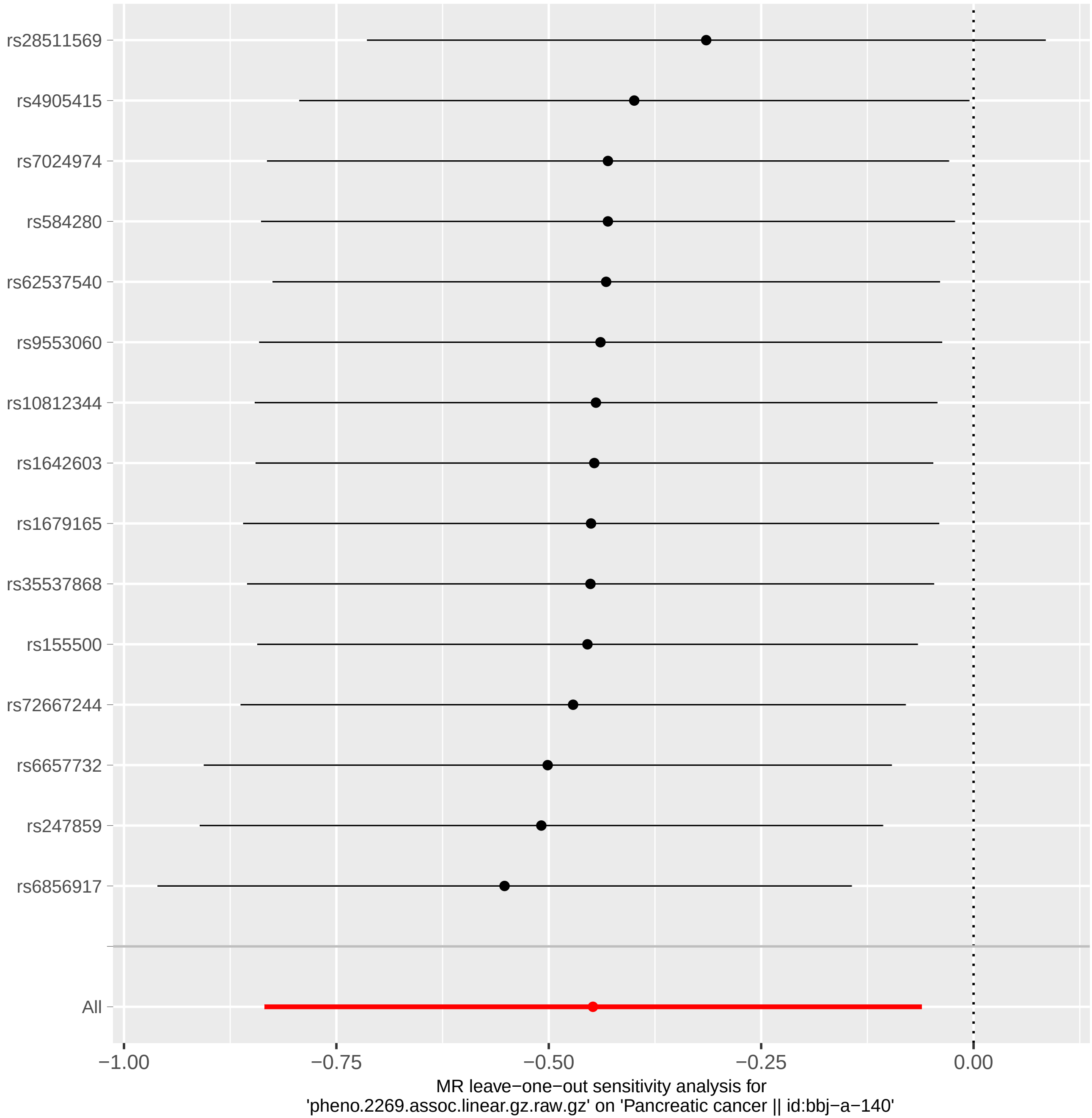
rs28511569
rs4905415
rs7024974
rs584280
rs62537540
rs9553060
rs10812344
rs1642603
rs1679165
rs35537868
rs155500
rs72667244
rs6657732
rs247859
rs6856917
All
−1.00
−0.75
−0.50
−0.25
0.00
MR leave−one−out sensitivity analysis for
'pheno.2269.assoc.linear.gz.raw.gz' on 'Pancreatic cancer || id:bbj−a−140'
